# Assessment of interobserver concordance in radiomic tools for lung nodule classification, with a focus on BRODERS and SILA

**DOI:** 10.1038/s41598-023-48567-7

**Published:** 2023-12-08

**Authors:** Faysal Al-Ghoula, Khushbu Patel, Sam Falde, Srinivasan Rajagopalan, Brian Bartholmai, Fabien Maldonado, Tobias Peikert

**Affiliations:** 1 Mayo Clinic, Rochester, USA; 2 Vanderbilt University Medical Center, Nashville, USA; 3 Nashville/Veteran Administration Tennessee Valley Health System, Nashville, USA

**Keywords:** Cancer screening, Non-small-cell lung cancer, Diagnostic markers

## Abstract

While CT lung cancer screening reduces lung cancer-specific mortality, there are remaining challenges. Radiomic tools promiss to address these challenges, however, they are subject to interobserver variability if semi-automated segmentation techniques are used. Herein we report interobserver variability for two validated radiomic tools, BRODERS (Benign versus aggRessive nODule Evaluation using Radiomic Stratification) and CANARY (Computer-Aided Nodule Assessment and Risk Yield). We retrospectively analyzed the CT images of 95 malignant lung nodules of the adenocarcinoma spectrum using BRODERS and CANARY. Cases were identified at Mayo Clinic (n = 45) and Vanderbilt University Medical Center and Nashville/Veteran Administration Tennessee Valley Health Care System (n = 50). Three observers with different training levels (medical student, internal medicine resident and thoracic radiology fellow) each performed lung nodule segmentation. All methods were carried out in accordance with relevant guidelines and regulations. Interclass correlation coefficients (ICC) of 0.77, 0.98 and 0.97 for the average nodule volume, BRODERS cancer probability and Score Indicative of Lesion Aggression (SILA) which summarizes the distribution of the CANARY exemplars indicated good to excellent reliability, respectively. The dice similarity coefficient was 0.79 and 0.81 for the data sets from the two institutions. BRODERS and CANARY are robust radiomics tools with excellent interobserver variability. These tools are simple and reliable regardless the observer/operator’s level of training.

## Introduction

Lung cancer screening by low dose chest CT scans (LDCT-LS) has been demonstrated to reduce lung cancer mortality by up to 20% in a high-risk population^1,2^. However, this benefit comes at the expense of many false positive results and the identification of clinically less concerning, indolent lung cancers (overdiagnosis) which have been estimated to affect > 90% of the screened individuals^3^. Considering that the estimated number of Americans who qualify for LDCT-LS is estimated to be > 8 million, as well as the ever-growing numbers of incidentally detected lung nodules due to widely available, technically advanced cross-sectional CT imaging of the chest^4^, the potential impact of the identification of benign nodules and indolent, clinically less concerning lung cancers is enormous. This highlights the urgent need for effective and ideally non-invasive biomarkers to facilitates the differentiation between benign and malignant lung nodules and the risk stratification of malignant nodules. Radiomics carries tremendous potential for the characterization of incidentally or screen-detected lung nodules. It provides a non-invasive method for extracting quantitative data from medical images, to aid with nodule identification and characterization to guide personalized lung nodule management^5,6^. While only a limited number of radiomic tools have been clinically validated, including, Lung Cancer Prediction (LCP) tool (Optellum), BRODERS (Benign vs aggressive nODule Evaluation using Radiomic Stratification), and CANARY (Computer Aided Nodule Assessment and Risk Yield), many other models are being developed. While LCP automates this process^7^, mitigating inter-segmenter variability, though it lacks a proofreading mechanism for comprehensive nodule inclusion most nodule characterization tools, including BRODERS and CANARY, critically depend on nodule segmentation to determine to input for the model.

We have previously demonstrated that the BRODERS can successfully differentiate benign from malignant lung nodules^8^. BRODERS is a predictive radiomics model that uses eight imaging features describing nodule location, size, shape, texture, and surface characteristics to differentiate Benign from malignant lung nodules^8^. In addition, we have previously developed and validated which analyzes lung nodules based on the distribution of 9 texture based, color coded exemplars. CANARY analysis allows the “virtual biopsy” and post-surgical outcome-based risk stratification of pulmonary nodules of the lung adenocarcinoma spectrum^9^. The distribution of the 9 CANARY exemplars can be summarized numerically by the continuous Score Indicative of Lung cancer Aggression (SILA score). The SILA score provides a quantitative approximation of the degree of histologic invasion and helps to predict the patient's survival^10^.

Both CANARY and BRODERS use a semi-automated approach, which requires the user to select the nodule location by seed placement and manually adjust the automatically selected boundaries of the nodule. These manual steps can theoretically affect the volume, surface characteristics, the distribution of the texture-based exemplars and SILA score. The potential impact of user-based variability may be particularly impactful for sub-solid lesions and in the presence of adjacent solid looking densities such as blood vessels, chest wall or mediastinal structures. It is possible that user-based manual adjustments to correct potential issues related to the automatic segmentation may result in significant interobserver variability which may affect the quantitative and qualitative results of the radiomics tools. This may result in nodule misclassification which can affect the performance of these tools in clinical practice.

We have previously demonstrated very good interobserver reliability for the CANARY based classification of pulmonary nodules of the lung adenocarcinoma spectrum^11^. However, the effects of semi-automated nodule segmentation on the results of the BRODERS model and the CANARY derived SILA score have not yet been explored. Herein we investigated the inter-operator variability of both the BRODERS model and the SILA score among three users at from 2 institutions (Mayo Clinic, Rochester, MN and Vanderbilt University, Nashville, TN) for a set of 95 indeterminate pulmonary nodules.

## Methods

This retrospective analysis was considered as minimal risk, and received an informed consent waiver from the Institutional Review Boards (IRBs) at Mayo Clinic (IRB number: 14-000666, Rochester, MN) and Vanderbilt University Medical Center (VUMC) (IRB numbers: 000616) and Nashville Veteran Administration Tennessee Valley Health System (TVHS) (IRB Number:030763). All methods were carried out in accordance with relevant guidelines and regulations. the experimental protocol was approved by the institutional review boards at Mayo Clinic hospital in Rochester and Vanderbilt university.

### Study subjects

In this retrospective study, cases were identified by reviewing electronic medical records and datasets of nodules at the three institutions: Mayo Clinic (n = 45), VUMC, and TVHS (n = 50). Our study included adult patients (> 18 years) who had adenocarcinoma spectrum malignant lung nodules measuring 6–30 mm, identified from non-contrast chest CT scans^8^. None of the patients were younger than 40 years old. The analyzed pulmonary nodules included: 54 solid nodules and 41 sub-solid nodules (33 part-solid and 8 pure ground glass nodules) The rationale for exclusively focusing on lung adenocarcinoma nodules is that CANARY had been developed and validated in lung adenocarcinomas and that these lesions can also be analyzed using BRODERS. To protect patient confidentiality, the CT scans were de-identified and shared between the participating institutions. Table 1 presents important demographic characteristics and tumor staging information for the analyzed cohort.

**Table 1:** Demographics and tumor staging for the studied cohort.

Demographics	Mayo clinic (N = 45)	VUMC/TVHS (N = 50)
Mean age at diagnosis	67	68.6
Male gender	37 (74%)	18(40%)
Smoking history		
Current	15 (30%)	8 (17%)
Former	31 (62%)	36 (80%)
Never	4 (8%)	6 (13%)
TNM stage		
IA	8 (16%)	5 (11%)
IB	2 (4%)	1 (2%)
IIA	2 (4%)	2 (4%)
IIB	1 (2%)	4 (9%)
IIIB	1 (2%)	1 (2%)
IV	0	0

Parentheses indicate variable percentages relative to total patients per institution.

### Image acquisition

The CT scans from the Mayo Clinic cohort in this study were obtained between 2009 and 2015. We analyzed indeterminate pulmonary nodules (IPNs) sized between 6 and 30 mm. The CT images were acquired using different types of scanners, such as GE Medical Systems LightSpeed Ultra, LightSpeed VCT, or LightSpeed Pro 16 (Waukesha, WI, USA); Toshiba Aquilion (Tustin, CA); Siemens Sensation 16 and Sensation 64 (Malvern, PA). The CT reconstruction algorithms varied by the scanner model and brand and included both high-resolution (lung) and smooth (soft tissue) filtered back-projection algorithms. The reconstructed images used a 512-matrix with slice thickness ranging from 1.25 to 2.5 mm, and the field of view was adjusted for each patient to cover the entire chest wall and both lungs.

The cohort from VUMC) and TVHS consisted of 50 patients aged over 18 years with IPN measuring between 6 and 30 mm. These nodules were identified through CT scans conducted between 2009 and 2015. All nodules were confirmed as adenocarcinoma (ADC) based on surgical resection. The TVHS CT scanner utilized was a 64-slice helical VCT from GE Medical Systems (Waukesha, WI, USA), while the VUMC CT scanner was an 8-slice helical STE scanner, also from GE Medical Systems. The CT imaging parameters for both scanners were set at 120 KVP, with automatic milliampere-second adjustments ranging from 30 to 400 to minimize radiation exposure. CT reconstruction algorithms varied depending on the scanner brand/model and included both smooth (soft tissue) and high-resolution (lung) filtered back-projection algorithms. The reconstructed images were generated using a 512-matrix with a slice thickness of 1.25–2.5 mm. The field of view was adjusted individually for each patient to encompass the entire chest wall and both lungs.

### CANARY and BRODERS observers

Authors ECN, MPF, and TFJ served as the three observers who evaluated CT images with suspicious lung nodules and performed CANARY analysis upon the cohort of 50 patients from VUMC/TVHS and 45 patients from Mayo. ECN, observer #1, was a second-year Internal Medicine resident at VUMC, and she had received introductory radiology training as a medical student. MPF, observer #2, was a fourth-year undergraduate student at Vanderbilt University and had no prior radiology training. TFJ, observer #3, was a thoracic radiology fellow in the sixth year of his radiology training.

### CANARY and BRODERS segmentation

**Figure 1:**
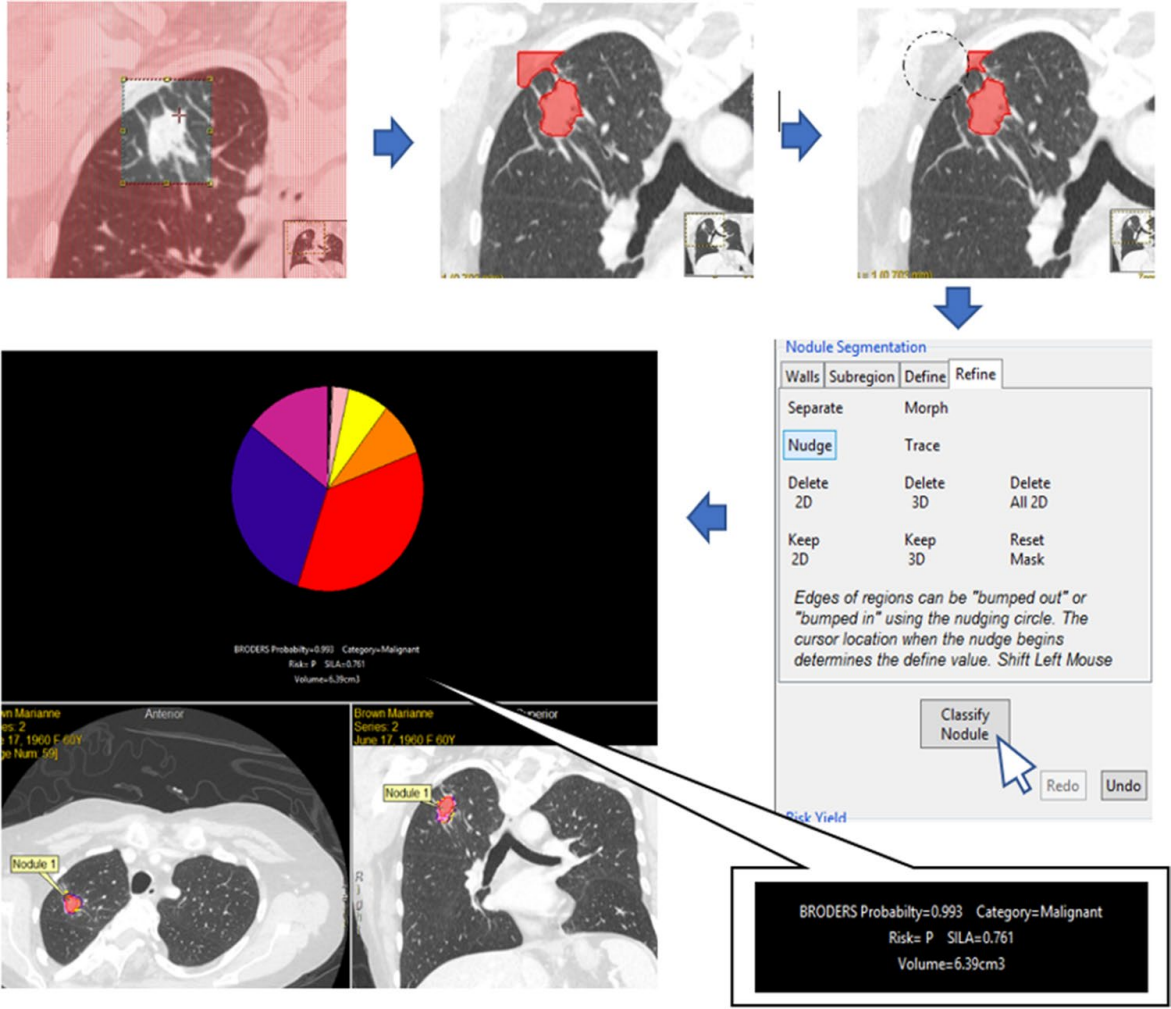
Overview of the CANARY segmentation process.

In the case of CANARY, after uploading the CT scans to the software. Nodule of interest was selected by the observers using the mouse to click on it. Subsequently, CANARY generated a partially automated "mask" within a predefined boundary on every individual slice of the CT scan. The borders of the selected nodule then fine-tuned by the observer to ensure that it accurately encompassed only the desired nodule. Once the observer confirmed or adjusted the borders, CANARY performed the nodule classification. Further elaboration on this method, including a detailed explanation, will be provided in the “Discussion” section^8^ (Fig. 1).

Regarding BRODERS, the nodules of interest underwent manual segmentation using the ANALYZE software developed by the Biomedical Imaging Resource at Mayo Clinic in Rochester, MN. We visually identified the location and size of each nodule. This was followed by the tracing of two-dimensional borders in alignment with the transverse orientation. To initiate the segmentation process, a semi-automated region-growing approach was employed, utilizing an operator-designed bounding cube that encompassed the nodule along with a seed location within the nodule. Subsequently, manual adjustment was undertaken to eliminate interfering structures such as vessels and pleura.

### Statistical analysis

The primary goal of this study was to assess the variability in SILA score and BRODERS model analysis between three different observers. Nodule volume, SILA score, and BRODERS probability were compared between observers’ segmentations. The variables mentioned above were assessed for variance to measure the deviation of each observer's segmentation from the mean of all three observers' segmentation. Intraclass Correlation Coefficient (ICC) was used to determine the ratio of nodule variance to the total variance, where the total variance comprises between-nodule variance, between-observer variance, and unexplained variation. When between-nodule variance increases, the contribution of between-observer variance to the total variance decreases. By employing a linear mixed effect model, the ICC was computed to assess the reproducibility of each voxel class identified in CT images of the pulmonary nodules. Furthermore, Dice similarity coefficient (DSC) was used to compare the segmentations similarity between any two observers for each examined nodule. The agreement of SILA and BRODERS characterizations for the lung nodule among the three observers was assessed using the Fleiss kappa coefficient.

## Results

**Figure 2:**
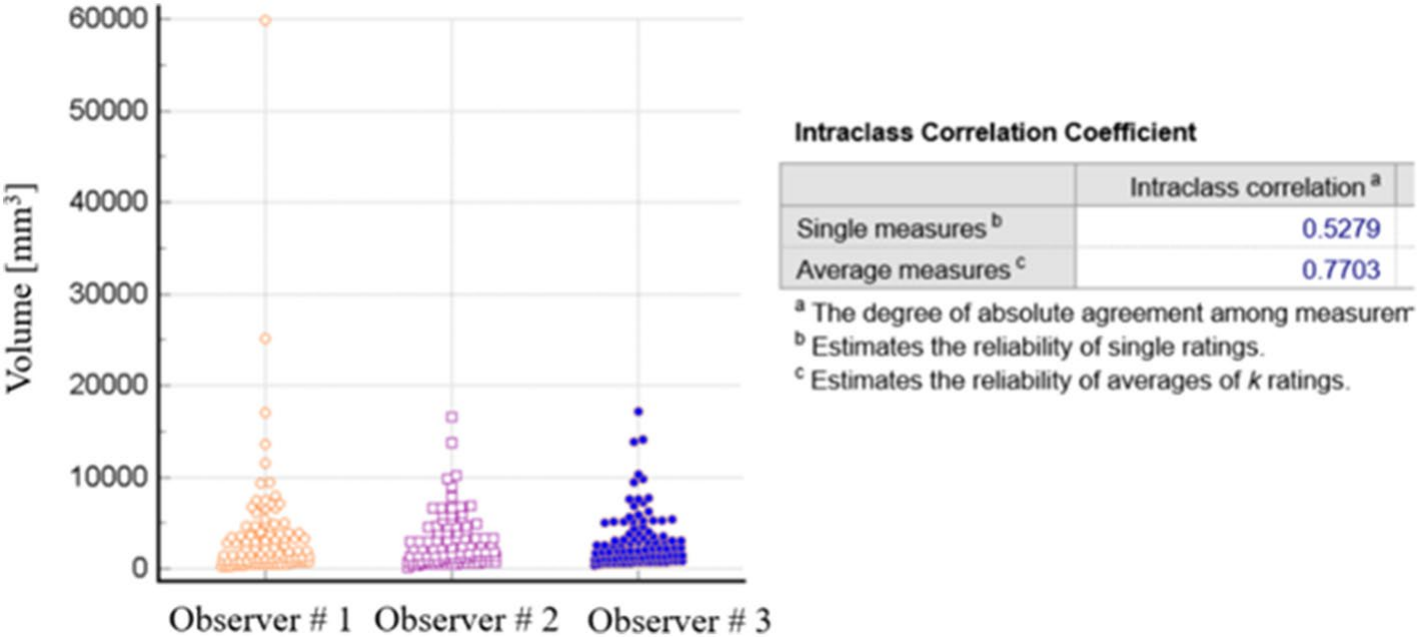
Interobserver agreement in measure nodule volume.

We used the interclass correlation coefficient (ICC) to compare the reliability of the measurements done by the 3 different observers (ECH (#1), MPF (#2) and TFJ (#3)) for the same lung nodules. Regarding the lung nodule volume, single measurements showed ICC of 0.52 indicating moderate reliability of a single observer. Average volume measurements between observers showed better ICC of 0.77 indicating good reliability (Fig. 2).

**Figure 3:**
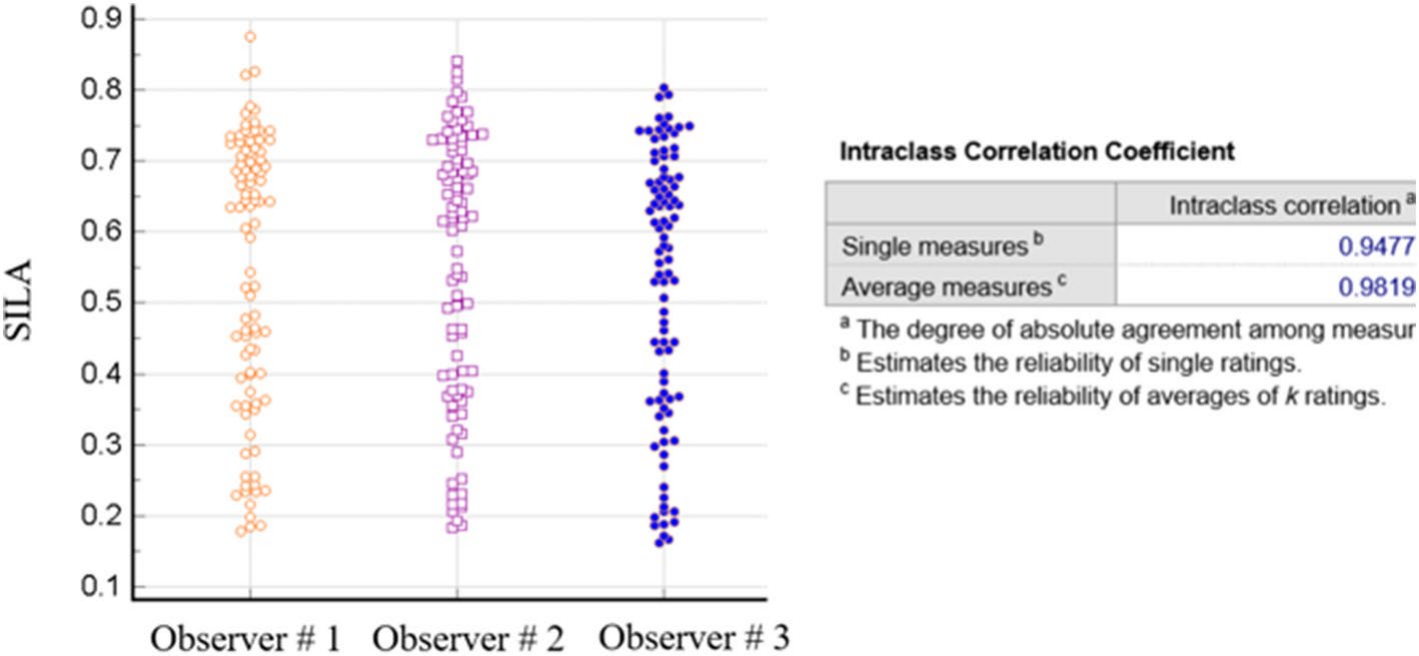
Interobserver agreement in measuring SILA.

Interestingly, CANARY, SILA, measurements showed improved ICC, with ICC of 0.94 for the single measures (ICC of > 0.9 indicates excellent reliability), and even better ICC for the pooled segmentations (Fig. 3).

**Figure 4:**
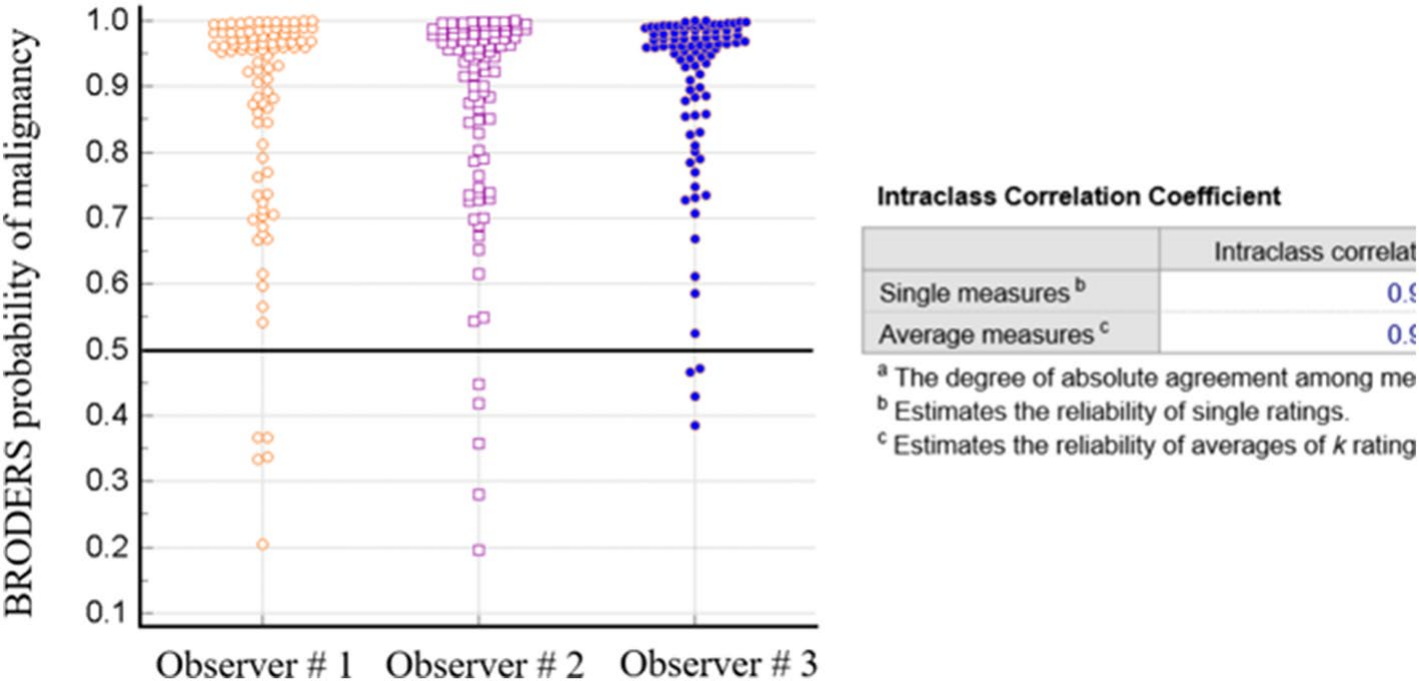
Interobserver agreement for the BRODERS probability of malignancy for the analyzed lung nodules.

Similarly, BRODERS probability showed consistent measurements with ICC of 0.93 with each single individual probability and the ICC of 0.97 with average measurements (Fig. 4). It is important to highlight that the cutoff between benign and malignant is 0.5 with BRODERS, and so 2 observer’s measurements identified 5 nodules as benign while the third identified 4 as benign with BRODERS probability close to 0.52 for the fifth nodule that was considered as malignant.

To evaluate the consistency in segmentation between the observers, Dice similarity coefficient (DSC) was calculated. Values > 0.7 indicate strong overlap and is used in segmentation validation literature as a threshold for acceptable reproducibility. For the VUMC/TVAH cases (n = 46) the mean DSC was 0.79 (standard deviation 0.13, 95% CI 0.77–0.81). For Mayo Clinic cases (n = 45) mean DSC was 0.81 (standard deviation 0.10, 95% CI 0.80–0.83). Four cases from VUMC/TVH were excluded due to failure to generate proper files required for DSC calculation.

## Discussion

The understanding of interobserver variability is crucial to the clinical application of new radiomic tools, such as BRODERS and CANARY, which involve semiautomated user-dependent steps.

CANARY analysis was developed using machine learning techniques based upon the analysis of the radiodensity of biopsy proven adenocarcinomas (with variable degrees of invasiveness)^9–12^. This process identified nine distinct clusters (exemplars) to represent the disease spectrum of lung adenocarcinoma. The distribution of these exemplars correlates well with the invasiveness of the lesion and provides an opportunity for non-invasive biopsy^9^. Furthermore, unsupervised clustering of the glyphs, representing a summary of the nine color coded exemplars (Violet (V), Indigo (I), Blue (B), Green (G), Yellow (Y), Orange (O), Red (R), Cyan (C), and Pink (P)) identified three patient groups with distinctly different post-surgical outcomes^13^. The Score Indicative of Lung Cancer Aggression (SILA) represents a cumulative aggregate of normalized distributions of the ranked CANARY exemplars. It creates a continues score that has been shown to correlate well with both the degree of histological tissue invasion and patient outcomes^10^. This opens many opportunities for the clinical application of CANARY to facilitate the noninvasive classification and individualized management of adenocarcinoma spectrum pulmonary nodules.

The BRODERS classifier/model was developed and internally validated using 726 lung nodules identified from the NLST dataset. A comprehensive set of quantitative radiomic features selected using LASSO regression were included for the development of a multivariable predictive model to discriminate benign from malignant lung nodules. The radiomics features included into BRODERS represent of different nodule characteristics: 1. Nodule location, 2. Nodule size, 3. Nodule shape, 4. Nodule radiodensity 5. Nodule texture, 6. Texture/radiodensity of the nodule-free surrounding lung, 7. Nodule surface characteristics and 8. Distribution of the nodule surface characteristics exemplars^11^. BRODERS was recently validated in an independent dataset where it outperformed the Brock model with the AUC was 0.87 (95% CI 0.81–0.92) for the Brock model and 0.90 (95% CI 0.85–0.94) for the BRODERS model^11^. This data highlights the potential clinical value of BRODERS to distinguish benign from malignant lung nodules.

However, both radiomics tool require the selection and semiautomated segmentation of the target lesion by a human user. The segmentation of the nodule is done by seed growing algorithm to create a “mask” that is verified and adjusted manually by the observer. Theoretically, many factors might affect the accuracy and reliability of radiomic tools. For focal lung lesions these factors include: the CT scanners manufacturer, different CT scan acquisition parameters, reconstruction algorisms, slice thickness and the presents and absence of intravascular contrast material. The good performance of both CANARY and BRODERS on the technically diverse multicenter NLST data set and additional external validation sets suggest that the performance of these tools is robust regarding these variables. In addition, we have previously specifically demonstrated that CT scanner type and the slice thickness have minimal impact on the reliability of the CANARY outcomes using the same dataset^11^. In this study we report the consistency of the performance of these tools, in particular, the interobserver agreement in nodule volume measurement, identifying SILA scores and BRODERS probabilities when used by 3 different observers from different institutions, with different backgrounds ranging from student level to radiology fellow level.

In accordance with prior studies, it was not surprising that we observed only moderate to good reliability for the segmented volumes of the lung nodules. This is not surprising given that the adjustments of the boundaries of the nodule is partially subjective and therefore it is almost impossible to have very accurate correlation of volume measurements. However, we clearly demonstrated that CANARY, specifically SILA, and BRODERS were not affected by this variability in the segmented nodule volume and demonstrated excellent interobserver reliability.

The findings of our study confirm that CANARY and BRODERS, including the semiautomated segmentation step, are reliable tools in assessing lung nodules, and that their performance is not affected by the observer’s background, level of training or minor inaccuracies of volume estimation. This help in the clinical implementation of these radiomic tools to aid the evaluation and management of the early lung cancer and provide reliable predictive scores to individualize the care to the patient without the need for unnecessary invasive diagnostic measures. These promising results represent the next step towards further prospective validation and clinical implementation of CANARY and BRODERS.

## Conclusion

CANARY (SILA score) and the BRODERS model are robust radiomics tools that can differentiate between lung nodules within the adenocarcinoma spectrum and with excellent interobserver agreement. These tools are simple and appear to be reliable regardless of the observer’s speciality or level of training.

## Data Availability

The datasets used and/or analyzed during the current study available from the corresponding author on reasonable request.
